# The longitudinal impact of an evidence‐based multiple family group intervention (*Amaka Amasanyufu*) on family cohesion among children in Uganda: Analysis of the cluster randomized SMART Africa‐Uganda scale‐up study (2016–2022)

**DOI:** 10.1111/famp.13007

**Published:** 2024-05-18

**Authors:** William Byansi, Ozge Sensoy Bahar, Latoya Small, Phionah Namatovu, Josephine Nabayinda, Joshua Kiyingi, Abel Mwebembezi, Gertrude Nakigozi, Kimberly Hoagwood, Mary M. McKay, Fred M. Ssewamala

**Affiliations:** ^1^ School of Social Work Boston College Chestnut Hill Massachusetts USA; ^2^ Brown School Washington University in St. Louis St. Louis Missouri USA; ^3^ Luskin School of Public Affairs University of California at Los Angeles Los Angeles California USA; ^4^ International Center for Child Health and Development Field Office Masaka Uganda; ^5^ Reach the Youth Uganda Kampala Uganda; ^6^ Rakai Health Sciences Program Rakai Uganda; ^7^ Grossman School of Medicine New York University New York City New York USA; ^8^ Vice Provost Office Washington University in St. Louis St. Louis Missouri USA

**Keywords:** family cohesion, family strengthening intervention, family wellbeing, multiple family groups, sub‐Saharan Africa

## Abstract

Family functioning plays a critical role in childhood disruptive behavior disorders (*The Family Journal*, 2003, **11**(1), 33–41; *Research in Nursing and Health*, 2016, **39**(4), 229–243). Yet, there is limited research on the impact of evidence‐based family strengthening interventions on improving family cohesion as a protective factor among children experiencing behavioral challenges. To address this gap, we analyzed data (*N* = 636) from the SMART Africa‐Uganda study (2016–2022), a cluster randomized clinical trial testing an evidence‐based family‐strengthening intervention called Amaka Amasanyufu (translated as "Happy Families" in the local language). Children aged 8–13 and their caregivers were recruited from 26 public primary schools that were randomized to: (1) control condition receiving generalized psychosocial literature (10 schools), (2) intervention delivered via parent peers (eight schools), and (3) intervention delivered via community healthcare workers (eight schools). Children completed the family cohesion questionnaire at baseline, 8 weeks, 16 weeks, and 6 months post‐intervention completion. The intervention effectiveness was evaluated via a three‐level logistic mixed effects model with pairwise comparisons across study conditions within each time point. Participants in the parent–peer intervention group had greater odds of being in the higher family cohesion group than participants in the control group at 8 weeks (OR = 3.24), 16 weeks (OR = 1.88) and 6 months (OR = 2.07). At 8 weeks, 16 weeks, and 6 months, participants in the community health worker group had 3.98, 2.08, and 1.79 times greater odds of being in the higher family cohesion group than participants in the control group, respectively. Our findings strengthen the evidence base for Amaka Amansayufu as an effective intervention that can be utilized in SSA to improve family cohesion in families with children experiencing behavioral challenges.

Mental health conditions, including emotional and behavioral disorders, are major causes of illness and disability among young people (WHO, 2020). Globally, it is estimated that 1 in 7 (14%) 10–19 year olds experience mental health conditions (WHO, 2020), yet these remain largely undetected and untreated. Behavioral disorders are more common among younger adolescents compared to older adolescents. For example, conduct disorder occurs among 3.6% of 10–14 year olds and 2.4% of 15–19 year olds (WHO, 2020). In Sub‐Saharan Africa (SSA), where children compose half the population, about one in every seven children are estimated to struggle with mental illness (Cortina et al., 2012).

In Uganda, children and adolescents comprise more than 50% of the population (Daumerie & Madsen, 2010). and the majority live in circumstances that put them at risk for developing mental health conditions, including high rates of chronic poverty (Daumerie & Madsen, 2010), domestic violence (Koenig et al., 2003), and physical violence against children (Naker, 2005). Studies from specialized treatment centers in Uganda suggest that one in 12 children suffers from conduct problems, and one in nine experiences attention deficit hyperactive disorder (ADHD) (Wamulugwa et al., 2017). A recent study conducted with 2434 school‐going children ages 8–13 in southwest Uganda, a region heavily impacted by poverty and HIV, found that 6% of the participants scored positive for oppositional defiant disorder (ODD) and 2% scored positive for conduct disorder (CD) (Kivumbi et al., 2019).

Mental health conditions have a long‐lasting negative impact if not addressed. Specifically, DBDs can impair daily functioning, adversely impacting academic performance (Liu et al., 2017; Sayal et al., 2015), family and peer relationships, and overall quality of life (Loeber et al., 2000). In addition, disruptive behavior disorders (DBDs) are also associated with serious physical health problems, unemployment, substance abuse, violence, criminal behaviors, and legal problems in adulthood (Biederman, 2005).

Research suggests that the family environment is important in developing disruptive behavior disorders in children (Stormshak et al., 2000; Vis et al., 2016). The family environment involves different aspects of family functioning, including family cohesion, communication, problem‐solving, conflict management, and attachment (Henderson et al., 2003; Leeman et al., 2016). In adolescence, youth with disruptive behaviors have been associated with an overall lack of parental warmth, high rates of conflict, and poor parental monitoring of youth (Dishion et al., 2002). Studies have investigated the relationship between externalizing behaviors and aspects of the family environment. A meta‐analysis of 53 studies assessing the relationship between various chronic conditions and family functioning in a large sample of children from Western countries found a negative relationship between problem behaviors and aspects of family functioning such as family adaptability, overall family relationships, and family expressiveness (Leeman et al., 2016). Therefore, understanding how family functioning relates to problem behaviors is critical for intervention development. Lower family cohesion was reported by mothers of children with conduct disorder (Lucia & Breslau, 2006). Mothers reported low cohesion was associated with children's externalizing problems and increased family conflict. Lower family cohesion and higher family conflict were associated with more externalizing problems (Lucia & Breslau, 2006). Similar reports of low family cohesion were reported by youth who were associated with delinquent behaviors (Matherne & Thomas, 2001). This suggests that healthy family functioning may help protect against children's externalizing behaviors.

Additionally, several studies have examined the link between family cohesion and externalizing problems (Coe et al., 2018; Li & Jhang, 2023; Lucia & Breslau, 2006). For instance, a longitudinal study among 823 children aged 6 and 11 found that family cohesion at 6 years was significantly associated with attention problems, and the relationship was stable from 6 to 11 years (Lucia & Breslau, 2006). Specifically, children in families with higher cohesion had fewer internalizing and attention problems than those with lower cohesion. In a separate study among preschoolers, family cohesion mediated the relationship between maternal relationship instability and children's externalizing problems, indicating that family cohesion decreased externalizing problems when maternal instability was high (Coe et al., 2018). These studies suggest that to mitigate and treat externalizing problems, it is critical to target interventions for vulnerable children and families that strengthen family functioning, including family cohesion.

While a few studies tested the impact of family interventions on mental health among children impacted by HIV or by war in sub‐Saharan Africa (Betancourt et al., 2014; Puffer et al., 2016), to our knowledge, no studies have examined the impact of evidence‐based family strengthening interventions on improving family cohesion as a protective factor among children experiencing behavioral challenges. Interventions that support and strengthen families to mitigate these stressors are urgently needed to effectively address and prevent DBDs among children in low‐resource settings, including Uganda, interventions that support and strengthen families to minimize these stressors are urgently needed. To this end, we implemented the SMART Africa‐Uganda study between 2016 and 2022 (Ssewamala, Bermudez, et al., 2018; Ssewamala, Sensoy Bahar, et al., 2018), which investigated the Amaka Amasanyufu intervention in school settings among families of children with ODD and other DBDs living in low‐resource settings. The effectiveness of this intervention in reducing oppositional defiant disorders and poor functioning in Ugandan children has been established (Brathwaite et al., 2022; Ssewamala et al., 2023).

Previous research has shown that interventions delivered by peers improve health outcomes. Specifically, parent peers who share common cultural backgrounds, language, and an in‐depth understanding of the difficulties associated with raising children with oppositional defiant disorders (ODDs) and other disruptive behavior disorders (DBDs) in Uganda play a crucial role. Parent‐peer facilitators can draw upon their personal experiences navigating behavioral issues with their children. This might foster mutual understanding and establish stronger emotional ties between the parent‐peer facilitators, and caregivers. This, in turn, could result in caregivers feeling less socially isolated (Obrochta et al., 2011), experiencing reduced self‐blame (Obrochta et al., 2011), gaining more optimism and confidence in handling children's behavioral challenges (Hoagwood et al., 2010), and being more empowered to take proactive measures (Kutash et al., 2011). These shifts in caregiver attitudes can potentially translate into improved behavioral outcomes among children. Hence, this study examines the impact of a multiple‐family group‐based family‐strengthening intervention on family cohesion among children with elevated behavioral challenges and their caregivers. To prevent discrimination, the team purposefully included families with children in the same age range who did not screen positive for DBDs in the intervention, even though it was designed for children with DBDs, including ODD (McKay et al., 2011). This was done given the high levels of mental health stigma in the study area (Ssebunnya et al., 2009). Consequently, our primary emphasis in this analysis was on children who obtained positive scores on at least one of three measures based on caregiver reports during the initial screening (*N* = 636). We examined three hypotheses based on the existing literature and theoretical framework guiding this intervention.

1. Children in the community health workers group would have a significant increase in family cohesion over time compared to children in the control condition;
2. Children in the parent peer group would have a significant increase in family cohesion over time compared to children in the control condition;
3. Children in the parent peer group would have better family cohesion compared to children in the community health workers group.

## METHODS

### Study design

The SMART Africa‐Uganda scale‐up study (2016–2022) examined a three‐arm cluster‐randomized controlled trial implemented in 26 public primary schools across five districts in Southwestern Uganda (Ssewamala, Sensoy Bahar, et al., 2018). If a school's student population was between 350 and 600, it was considered for inclusion in the study. Additionally, schools were matched based on performance in national exams, student population, and geographical location. After school leaders indicated an interest in participating during introduction meetings, a list of 42 eligible schools was compiled. Thirty schools were chosen randomly from this list. The research team randomly assigned entire schools to the following study conditions using SPSS software: (1) A bolstered standard of care (BSOC) control group (*n* = 10 schools). The control group received material about mental healthcare and support for children with behavioral difficulties. This was supplemented by school notebooks and textbooks; (2) Multiple family group intervention delivered by parent peers (MFG‐PP; *n* = 10 schools); and (3) Multiple family group intervention delivered by community health workers MFG‐CHW (*n* = 10 schools). Both treatment groups also received BSOC. Because of COVID‐19 lockdowns around the country, baseline assessments and the intervention were not conducted in four schools (two from each treatment group (MFG‐PP; and MFG‐CHW), leaving eight schools for MFG‐PP; and eight schools for MFG‐CHWs). At the onset of COVID‐19 in Uganda, the baseline assessments and intervention delivery had not yet commenced in four treatment schools (two parent peers and two community health workers). The pandemic prompted a series of restrictions, including the closure of all educational institutions, the shift to online teaching, the prohibition of large gatherings, curfew enforcement, limitations on public transport and restrictions on inter‐district and intra‐district travel. These collective restrictions impeded the execution of research activities, preventing the research team from initiating baseline assessments and conducting multiple family group sessions in the remaining four treatment schools. Moving forward with the research activities would have endangered the wellbeing of the study participants and the field research team. This decision was approved by the Institutional Review Boards in Uganda, Washington University in St. Louis, and the funding body, the National Institute of Mental Health. We have published these methods and primary outcomes using this same approach and note this approved amendment to the study (Brathwaite et al., 2022; Ssewamala et al., 2023). Our description of the design and impact of field conditions is consistent across papers. The statistical power for this study was recalculated, and we retained sufficient statistical power to detect a small standardized mean difference (Brathwaite et al., 2022).

### Description of Amaka Amasanyufu MFG intervention

The 16‐session manualized Amaka Amasanyufu (Happy Families in Luganda, the local language in the study region) intervention was adapted (Sensoy Bahar et al., 2020) to the Ugandan context from the evidence‐based 4Rs and 2Ss intervention designed to strengthen six key family principles. The 4Rs, known as Rules, Responsibilities, Relationships and Respectful Communication, address parenting factors, including family organization, discipline practices, family connectedness, support, and communication. The 2Ss known as Stress and Social Support, focus on parenting stress and other life stressors and when and how to tap into support systems (Sensoy Bahar et al., 2020; Ssewamala, Bermudez, et al., 2018; Ssewamala, Sensoy Bahar, et al., 2018). The multiple family groups included 10–20 families, with at least two generations of a family present in each session, including caregivers/guardians, siblings, or other extended family members where applicable. The sessions, delivered in schools, lasted between 60 and 90 min and involved group discussions, role plays and other activities that facilitated support, learning, and interaction between the family members and among the families in the group (McKay et al., 2004).

Amaka Amasanyufu was delivered by parent peer or community health worker facilitators over 16 sessions, promoting task‐shifting (Sensoy Bahar et al., 2020). This strategy embraced task shifting, an important approach used to enhance mental health care delivery in settings where adequately trained health professionals are scarce. Task‐shifting entails assigning tasks from highly trained providers to persons with less training, such as community healthcare workers and parent peers (Hoeft et al., 2018). The Amaka Amasanyufu intervention, facilitated by either two‐parent peers or two community health workers, took place within school settings. The sessions typically occurred during school breaks on regular days, occasionally extending to weekends if there were conflicts with exams or other events during the week. Facilitator training was conducted separately for parent peers and community health workers to ensure effective implementation. The training was led by the team members who had received training from the study's principal investigator, the developer of the original intervention. The training emphasized both the content and the skills required for facilitation. To assess fidelity, facilitators completed the Knowledge Skills and Attitude Test (KSAT) (McKay et al., 2011). The assessment ensured their proficiency and mastery of the program's content, with a required pass score of 85%. Throughout the intervention, trainers were present during all sessions, observing and providing feedback to facilitators after each session.

Multiple groups and family theories inform the Amaka Amasanyufu, including psychoeducation and social group work, family systems theory, structural family theory, and social learning theory (McKay et al., 2011). In randomized control trials, the MFG intervention has been adapted and deployed among vulnerable adolescents (McKay et al., 2014; Mellins et al., 2014; Ssewamala et al., 2023), with findings indicating improved family processes (support, communication, and parent–child participation), adolescent mental health, self‐esteem, and reduced risk behaviors. Details on the theoretical frameworks guiding the cultural adaptation of the *4Rs and 2Ss* to *Amaka Amasanyufu* can be found in the adaptation paper that has been previously published (Sensoy Bahar et al., 2020).

### Participants

**FIGURE 1:**
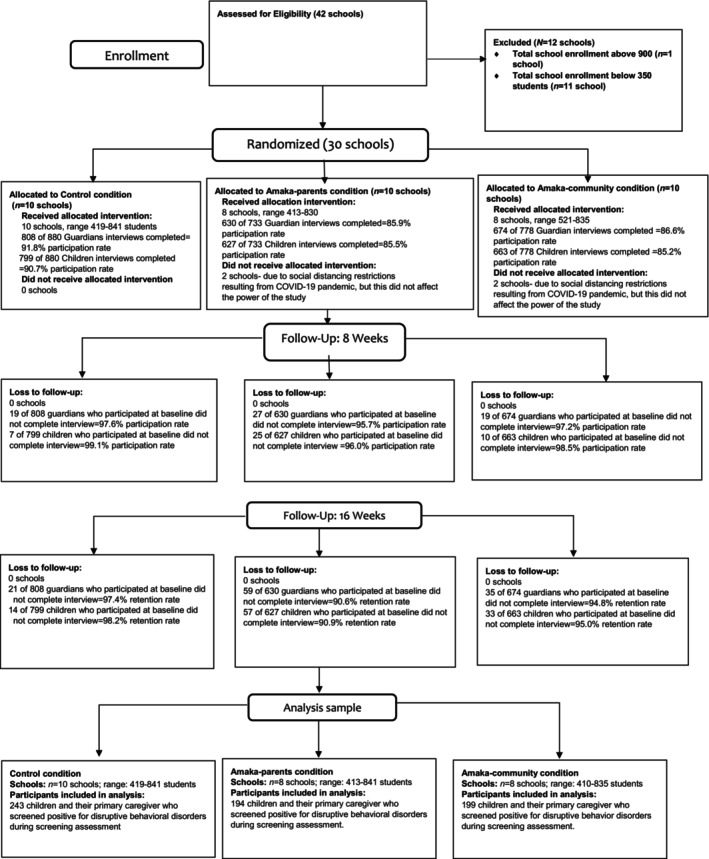
CONSORT flow diagram for the SMART Africa‐Uganda study.

Participants who met the following criteria were recruited in each school after randomization: (1) child/adolescent aged 8–13 in grades 2–7; (2) caregiver completed three screening measures for behavioral disorders in children/adolescents (Disruptive Behavior Disorder Rating Scale (Pelham et al., 2005), Iowa Conner's Scale (Waschbusch & Willoughby, 2008), and Impairment Rating Scale (Fabiano et al., 2006)); and (3) caregiver provided written consent and child/adolescent supplied assent to participate. If a child or adolescent scored positive on at least one of the screening tests, they were considered to have symptoms of disruptive behaviors (see study protocol (Ssewamala, Bermudez, et al., 2018; Ssewamala, Sensoy Bahar, et al., 2018) for more information). Children/adolescents and caregivers completed assessments at the start of the intervention (baseline) at 8 weeks (mid‐point), 16 weeks (end of intervention), and 6 months after the end of the intervention. Considering the prevailing negative perceptions surrounding mental health in the study region (Ssebunnya et al., 2009), the study enrolled all eligible children and their caregivers. This approach was adopted to prevent exacerbating the existing stigma and potential labeling of the children. The focus of our analysis in this paper is restricted to the subset of children who were identified with elevated symptoms of disruptive behavior disorders (DBDs) during the initial screening phase. All participants were blinded to the intervention arm. Refer to Figure 1 for the Consolidated Standards of Reporting Trials (CONSORT) flow diagram for the cluster‐randomized controlled trial phases.

### Ethics

The SMART Africa‐Uganda study received ethical approval from Washington University in St. Louis's Institutional Review Board (#2016011088), the Uganda Virus Research Institute (GC/127/16/05/555), and the Uganda National Council of Science and Technology (SS4090). The Data Safety and Monitoring Board at the National Institute of Mental Health also approved and monitored study procedures. Written informed consent/assent was obtained from all study participants. Participants were reimbursed for their time for completing assessments at each time point. Furthermore, to support family attendance for the Amaka Amasanyufu, families were reimbursed for transportation costs associated with attending each session.

### Outcome variable

The measures were administered in the local language (Luganda). Before administration, all measures were translated into Luganda, back‐translated into English, and pilot‐tested with native speakers to ensure comprehensibility and adapted for cultural appropriateness. In addition, the six‐item family cohesion scale has been used previously in other Suubi studies (Ssewamala et al., 2019; Ssewamala, Bermudez, et al., 2018; Ssewamala, Sensoy Bahar, et al., 2018) conducted among youth populations in Uganda.

### Family cohesion

Six items from the family relations scale were used to assess family cohesion (adapted from the parent–child relationship inventory, child caregiver communication scale, family assessment measure, and family adaptability and cohesion scale; Moos, 1994). The scale was assessed on a 5‐point Likert scale, with 1 = never occurs to 5 = always occurs. The children were asked to rate the frequency with which each item occurred in their family. The scale items include (1) Do your family members ask each other for help before asking non‐family members for help? (2) Do your family members like to spend their free time with each other? (3) Do your family members feel close to each other? (4) Are you available when others in the family want to talk to you? (5) Do you listen to what other family members have to say, even when you disagree? (6) We do things together as a family. At baseline, the scale had an acceptable internal consistency (Cronbach's alpha of 0.73). Given the limited research on family cohesion in this global context, we used the median split (“low” and “high”) at the baseline and follow‐up. Those above the median had higher family cohesion, and those below the median had lower family cohesion at baseline. Drawing from existing literature on family (Marsiglia et al., 2009; Rajesh et al., 2015; Wilkinson et al., 2011), high family cohesion served as a reference category because this quality has been found to be protective against DBDs.

### Data analysis

Summary statistics for the outcome by study group were computed at each time point. To estimate the impact of the intervention on family cohesion, a three‐level model was fit using a logistic GLMM via the Stata—melogit—command with the odds ratio reported to represent the change in odds of the outcome per unit change in the predictor. In this study, participants were nested within each school, and multiple observations over time were nested within each individual. Therefore, multilevel models have the advantage of being statistically efficient in accounting for the clustering pattern of data. Children from the same school are more likely to be correlated, and repeated measures for each participant are unlikely to be independent.

**FIGURE 2:**
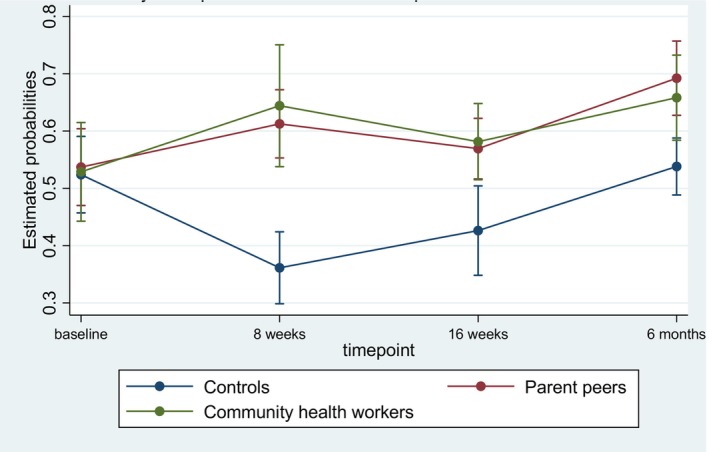
Adjusted probabilities for high family cohesion by study group and time with 95% confidence intervals.

The model comprised the fixed categorical effects for the study group (parent peers and community health workers with the control group serving as the reference group), time dummies (8 weeks, 16 weeks, and 6 months with baseline as the reference), their interactions, and random intercepts at the school level and person level. The unstructured covariance structure was modeled, which made the least assumptions regarding the covariance structure. To account for clustering, the model was estimated using Huber‐White standard errors (White, 1980). We used the contrast command in Stata to decompose omnibus effects for study group, time, and their interaction and group comparisons within each time point. Figure 2 provides a Margins plot to help describe the patterns of results observed. All analyses were conducted in Stata version 15.

## RESULTS

### Participant socio‐demographic characteristics

Participants socio‐demographic characteristics are presented in Table 1. At baseline, 636 participants evidenced symptoms of disruptive behavioral disorders. Additionally, 243 were randomly assigned to the control, 194 to the intervention group delivered by parent peers, and 199 to the intervention group delivered by community health workers. On average, participants were 11 years old, more than half (51.4%) were females, and 12% had lost both parents. Most participants (69%) reported their biological father or mother as their primary caregiver. The retention rate at 6‐month follow‐up was 93.4%. We used the *F*‐test and Wald chi‐square tests, adjusting for clustering within schools to test for group differences at baseline (Table S1A). However, none of the group differences were significant at baseline. The summary statistics for family cohesion by study group across time points are also presented in Table S1.

**TABLE 1:** Description of socio‐demographic characteristics of children with behavioral disorders at baseline.

Characteristics	Control *N* = 243	Parent peers *N* = 194	Community health workers *N* = 199	Total *N* = 636
Children's characteristics				
Age (years), Mean (SD)	10.9 (1.4)	11.6 (1.3)	11.8 (1.3)	11.4 (1.4)
Gender, *n* (%)				
Male	113 (46.5)	94 (48.5)	102 (51.3)	309 (48.6)
Female	130 (53.5)	100 (51.6)	97 (48.7)	327 (51.4)
Orphanhood status, *n* (%)				
Double orphan	2 (0.8)	4 (2.1)	5 (2.5)	11 (1.7)
Single orphan	37 (15.3)	24 (12.4)	20 (10.1)	81 (12.8)
Non‐orphan	203 (83.50	165 (85.5)	168 (84.9)	536 (84.3)
Missing	1 (0.4)	1 (0.5)	6 (3.0)	8 (1.3)
Primary caregiver, *n* (%)				
Biological parents	170 (70.0)	123 (63.4)	146 (73.4)	439 (69.0)
Grandparents	51 (21.0)	47 (24.2)	37 (18.6)	135 (21.2)
Other relatives	22 (9.1)	24 (12.4)	16 (8.1)	62 (9.8)

### The effect of the multiple family group intervention on family cohesion among children with elevated symptoms of disruptive behavior disorders

The odds ratio and test statistics from the mixed logistic model appear in Table 3. There was a statistically significant overall main effect of condition (χ^2^[2] = 22.61, *p* < 0.001) and a significant overall effect of time (χ^2^[2] = 17.98, *p* < 0.001). Moreover, these main effects were qualified by a statistically significant interaction between condition and time (χ^2^[2] = 27.23, *p* < 0.001).

### Group‐within‐time simple effects at 6‐month post‐intervention

As presented in Tables 2 and 3, participants in the parent peer intervention group had greater odds of being in the higher family cohesion group than participants in the control group at 8 weeks (OR = 3.24; 95%CI: 1.66, 6.34), 16 weeks (OR = 1.88; 95%CI: 1.17, 3.03), and 6 months (OR = 2.07; 95%CI: 1.15, 3.71; Table 3). Similarly, at 8 weeks, 16 weeks, and 6 months, participants in the community health worker group had 3.98, 2.08, and 1.79 times greater odds of being in the higher family cohesion group than participants in the control group, respectively (Table 3). However, parent peer and community health worker groups were not significantly different at any time point. At 6‐month follow‐up, the community health workers' intervention group was not statistically different from the control group. Taken together, the results indicate that all three groups experienced substantial improvements in family cohesion. However, the improvements were greater for the two intervention arms (Figure 2).

**TABLE 2:** Comparisons of study group probabilities within each time point for family cohesion (group‐within‐time simple effects).

Timepoint	Comparison	Family cohesion
		OR (95% CI)
8‐weeks	Parent peers versus Control	1.24 (0.77, 1.71)
	CHWs versus Control	1.4 (0.73, 2.08)
16‐weeks	Parent peers versus Control	0.7 (0.22, 1.17)
	CHWs versus Control	0.76 (0.24, 1.28)
6‐months	Parent peers versus Control	0.79 (0.35, 1.23)
	CHWs versus Control	0.61 (0.14, 1.07)
No. of participants	636	
No. of observations	2429	

**TABLE 3:** Odds ratio for logistic regression model and 95% confidence intervals for family cohesion.

Model components	Family cohesion (≥50th percentile = high family cohesion)	*p*‐value
	OR (95% CI)	
Study group χ^2^ (df)	**χ** ^**2**^ **(2) = 22.61**	**0.001**
Control group (ref category)		
Parent peers	1.07 (0.67, 1.70)	0.79
Community health workers (CHWs)	1.02 (0.60, 1.75)	0.93
Time, χ^2^ (df)	**χ** ^**2**^ **(3) = 17.98**	**0.001**
Baseline (ref category)		
8 weeks	**0.45 (0.32, 0.63)**	**0.001**
16 weeks	**0.62 (0.46, 0.84)**	**0.01**
6 months	1.07 (0.67, 1.70)	0.77
Group#Time, χ^2^ (df)	**χ** ^**2**^ **(6) = 27.23**	**0.001**
8 weeks# Parent peers	**3.24 (1.66, 6.34)**	**0.001**
8 Weeks# CHWs	**3.98 (2.24, 7.06)**	**0.001**
16 weeks# Parent peers	**1.88 (1.17, 3.03)**	**0.01**
16 Weeks# CHWs	**2.08 (1.13, 3.82)**	**0.02**
6 months# Parent peers	**2.07 (1.15, 3.71)**	**0.02**
6 months# CHWs	1.79 (0.84, 3.80)	0.13
Constant	1.12 (0.81, 1.55)	0.48
ICC (95% CI): Schools	0.004 (<0.001, 0.103)	
ICC (95% CI): Participants	0.234 (0.165, 0.322)	
No of participants	636	
No of observations	2429	

*Note*: For categorical outcome, the model comprised the outcome along with fixed categorical effects for the study group (control vs. Parent peers vs. CHWs), time (baseline, 8 weeks, 16 weeks, and 6 months), a group‐by‐time interaction term, and random intercepts at the school‐ and person‐level with robust standard errors. Bolded numbers are significant.

Abbreviations: CI, confidence interval; ICC, intraclass correlation coefficient.

## DISCUSSION

The study examined the impact of Amaka Amasanyufu, a multiple family group family strengthening intervention, on family cohesion/relationships among a sample of children experiencing elevated symptoms of DBDs in Uganda. The results showed that participation in Amaka Amasanyufu significantly improved family cohesion among children exhibiting elevated symptoms of behavioral problems. Specifically, both intervention groups, facilitated by community healthcare workers and parent peers, experienced significant improvements in family cohesion compared to the control condition. Family cohesion is essential for building an emotional bond that family members have with each other (Coe et al., 2018). This enhances support and ultimately strengthens both families' ability to deal with external stressors and emotional and behavioral responses to the behavioral needs of children and adolescents (McKay et al., 2004).

Importantly, in low‐resource settings, research shows that children and adolescents with DBDs are likely to have comorbidity with mood disorders, including depression (Patel et al., 2018), and those with co‐morbid DBDs and depression have a greater burden of functional impairment (Copeland et al., 2007; Marmorstein & Iacono, 2004; McCrone et al., 2005). Furthermore, DBDs place a substantial strain on caregivers' mental health, with one study showing maternal caregiver strain was independently predicted by having a child with DBD (Bussing et al., 2003). Consequently, DBDs are a major contributor to parental stress (Evans et al., 2009), which influences caregivers' ability to cope and effectively manage these children in low‐resource communities, including those in southwestern Uganda, where families endure several poverty‐related circumstances that further exacerbate parental stress (Ministry of Gender, 2015), all of which are risk factors undermining family cohesion.

This is particularly important as family cohesion has been documented as a protective factor against child behavioral challenges and several other mental health and health challenges (Daniels & Bryan, 2021). Previous research from Uganda, particularly among children and adolescents orphaned by HIV and those living with HIV and AIDS, has shown an association between family cohesion and a wide range of youth outcomes, including HIV treatment adherence and mental health (Damulira et al., 2019; Nabunya et al., 2020; Nyoni et al., 2019). For instance, youth in Uganda reported lower levels of depression and higher self‐concept when living in a cohesive family (Nyoni et al., 2019). Higher family cohesion was associated with increased ART adherence (Damulira et al., 2019). Families in sub‐Saharan Africa rely on a tight network of kinship and non‐kinship relations for members' support, loyalty, and exchange (Lwelunmor et al., 2006). In the Uganda setting, this includes providing instrumental and emotional support that is essential for the wellbeing of children and adolescents (Nabunya et al., 2019). Additionally, cohesive families are much more likely to be aware of and assist children and adolescents experiencing behavioral needs.

Additionally, higher family cohesion is associated with lower adolescent alcohol use, psychological stress, and delinquent behaviors. This cohesion can also mitigate the effects of caregiver alcohol consumption against negative adolescent behaviors (Zimmerman & Farrell, 2017). Not only does family cohesion buffer the effects of stressful life events against adolescent behavioral outcomes, it can also strengthen the family's ability to cope with external stressors (Rotheram‐Borus et al., 2005). Hence, implementing family interventions that bolster family cohesion may effectively improve behavioral and mental health outcomes for children and caregivers in families where children struggle behaviorally.

Although the intervention arms did better than the control at 8 and 16 weeks, at 6‐month no significant difference was observed between the community health workers' intervention group and the control group. In addition, the control group also showed a trend toward better family cohesion, though the difference was not statistically significant. It is possible that providing caregivers with mental health materials may have eased some of the caregiver concerns around their child's behaviors and improved child–caregiver relationships to a certain extent, though not sufficiently. It is also possible that parent peers may have related to the content and conveyed it more effectively to participating families better, given that they also identified themselves as parents, and hence helped the families in that intervention group to sustain improvements in family cohesion at a significant level at 6 months when compared to the control group. Alternatively, it is possible that parent peers and families from those same communities continued to have conversations after the intervention ended, allowing families to sustain the progress made. Hence, further research is needed.

Our results should be interpreted in light of the following limitations. First, due to the use of self‐reported data, there is a chance that participants gave socially acceptable responses. Second, the intervention was developed to address child behavioral outcomes and not specifically family cohesion. However, several aspects of the intervention targeted aspects of family life that can strengthen family cohesion, including respectful communication, relationships, rules, and responsibilities. The 6‐month post‐intervention follow‐up period is also considered to be medium term. We cannot assess whether this intervention increased family cohesion over the longer term. Additionally, obtaining information from multiple sources, including teachers and caregiver reports, would help triangulate the data. This is especially important because DBDs were solely identified based on reports from caregivers. Our study is based upon significant previous findings from this dataset that it is important for interventions to positively impact and strengthen family processes, such as family cohesion (Brathwaite et al., 2022; Byansi et al., 2022; Ssewamala et al., 2023). This finding is consistent with existing literature, and we recommend that future intervention studies be strengthened by obtaining information from multiple sources, including children and other family members. The inclusion of multiple reporters and a more comprehensive mental health evaluation among those with elevated symptoms of DBDs are important next steps. Similarly, future research should examine how family cohesion mediates targeted child outcomes in psychosocial interventions. The COVID‐19 pandemic notably influenced the design of this study. The unprecedented global health crisis restricted our methodological choices, particularly regarding participant recruitment and data collection methods. However, despite these constraints, careful measures were taken to ensure the robustness of the study's outcomes. We assessed baseline differences between the control and treatment arms to identify potential disparities, and no significant differences were observed.

Despite these limitations, the study results have important practice and policy implications. The Amaka Amasanyufu MFG intervention is a promising family‐focused intervention that could be delivered by both parent peers and community health workers, opening up opportunities for making these interventions more accessible at low‐resource settings to those families who are in need, given the dearth of clinically trained mental health staff in Uganda as well as in SSA in general (WHO, 2016). This is crucial because it offers a chance to increase the lay workforce's capacity to support caregivers with community‐level mental health interventions and reduce the burden on an already stretched healthcare system (Galvin & Byansi, 2020; Purgato et al., 2020; Seidman & Atun, 2017). In addition, the intervention was successfully delivered in school settings. Hence, providing government support for this intervention to be incorporated and scaled up in the education system may be considered.

In light of this, future studies should examine the different mechanisms through which family cohesion impacts the relationship between multiple family group interventions and children's behavioral outcomes. Additionally, it is important to longitudinally examine how improved family cohesion impacts caregivers' mental health and emotional wellbeing, especially for those in low‐resource settings. Finally, future research should continue to examine the potential impact of the different lay workforce as facilitators of the intervention on targeted outcomes.

## CONCLUSIONS

The Amaka Amasanyufu MFG intervention significantly improved family cohesion compared to the usual care among caregivers of children with reported DBDs in low‐resource communities in Uganda. Many intervention studies do not evaluate the impact of family cohesion despite its usefulness in various mental health outcomes. Hence, our findings strengthen the evidence base for the Amaka Amansayufu MFG intervention being an effective intervention that can be utilized and potentially scaled up across other low‐resource communities in SSA to simultaneously improve family cohesion among caregivers and reduce/prevent DBDs among children and adolescents.

## Supporting information


Data S1.
