# Supplementary material for: The longitudinal impact of an evidence‐based multiple family group intervention (Amaka Amasanyufu) on family cohesion among children in Uganda: Analysis of the cluster randomized SMART Africa‐Uganda scale‐up study (2016–2022)
Source: Fam Process. 2024 May 18;63(4):1851–66. doi: 10.1111/famp.13007 (PMC11659092; doi:10.1111/famp.13007)
Supplement: Supplementary file 1 — Data S1. [file FAMP-63-1851-s001.docx]

| **Outcome** | **Study arm** | **Category** | **Baseline** | | **8 weeks** | | **16 weeks** | | **6 months** | |
| --- | --- | --- | --- | --- | --- | --- | --- | --- | --- | --- |
|  |  |  | **N** | **n (%)** | **N** | **n (%)** | **N** | **n (%)** | **N** | **n (%)** |
| Family cohesion | Control | High family cohesion | 243 | 127 (52.26) | 235 | 85 (36.17) | 235 | 100 (42.55) | 223 | 119 (53.36) |
|  |  | Low family cohesion |  | 116 (47.74) |  | 150 (63.83) |  | 135 (57.45) |  | 104 (46.64) |
|  | Parent peers | High family cohesion | 194 | 104 (53.61) | 186 | 114 (61.29) | 173 | 99 (57.23) | 179 | 125 (69.83) |
|  |  | Low family cohesion |  | 90 (46.39) |  | 72 (38.71) |  | 74 (42.77) |  | 54 (30.17) |
|  | Community health workers | High family cohesion | 199 | 105 (52.76) | 193 | 124 (64.25) | 185 | 107 (57.84) | 185 | 121 (65.41) |
|  |  | Low family cohesion |  | 94 (47.24) |  | 69 (35.75) |  | 78 (42.16) |  | 64 (34.59) |

**Supplementary Table S1**

*Summary of the Outcome by Study Group and Time Point*

| **Characteristics** | **Control**  **N=243** | **Parent peers**  **N=194** | **Community health workers**  **N=199** | **F-test or Wald Chi-Square** |
| --- | --- | --- | --- | --- |
| **CHILDREN’S CHARACTERISTICS** |  |  |  |  |
| **Age (years), Mean (SD)** | 10.9 (1.4) | 11.6 (1.3) | 11.8 (1.3) | 1.05, P=0.36 |
| **Gender, n (%)** |  |  |  | 0.22, P=0.90 |
| Male | 113 (46.5) | 94 (48.5) | 102 (51.3) |  |
| Female | 130 (53.5) | 100 (51.6) | 97 (48.7) |  |
| **Orphanhood status, n (%)** |  |  |  | 3.09, P=0.54 |
| Double orphan | 2 (0.8) | 4 (2.1) | 5 (2.5) |  |
| Single orphan | 37 (15.3) | 24 (12.4) | 20 (10.1) |  |
| Non-orphan | 203 (83.50 | 165 (85.5) | 168 (84.9) |  |
| Missing | 1 (0.4) | 1 (0.5) | 6 (3.0) |  |
| **Primary caregiver, n (%)** |  |  |  | 5.84, P=0.21 |
| Biological parents | 170 (70.0) | 123 (63.4) | 146 (73.4) |  |
| Grandparents | 51 (21.0) | 47 (24.2) | 37 (18.6) |  |
| Other relatives | 22 (9.1) | 24 (12.4) | 16 (8.1) |  |

**Supplemental Table 1A.**

*Description of Socio-demographic Characteristics of Children with Behavioral Disorders at Baseline by Group Assignment*
